# Integrating Evolution into Ecological Modelling: Accommodating Phenotypic Changes in Agent Based Models

**DOI:** 10.1371/journal.pone.0071125

**Published:** 2013-08-05

**Authors:** Aristides Moustakas, Matthew R. Evans

**Affiliations:** School of Biological and Chemical Sciences, Queen Mary, University of London, London, United Kingdom; Université de Nantes, France

## Abstract

Evolutionary change is a characteristic of living organisms and forms one of the ways in which species adapt to changed conditions. However, most ecological models do not incorporate this ubiquitous phenomenon. We have developed a model that takes a ‘phenotypic gambit’ approach and focuses on changes in the frequency of phenotypes (which differ in timing of breeding and fecundity) within a population, using, as an example, seasonal breeding. Fitness per phenotype calculated as the individual’s contribution to population growth on an annual basis coincide with the population dynamics per phenotype. Simplified model variants were explored to examine whether the complexity included in the model is justified. Outputs from the spatially implicit model underestimated the number of individuals across all phenotypes. When no phenotype transitions are included (i.e. offspring always inherit their parent’s phenotype) numbers of all individuals are always underestimated. We conclude that by using a phenotypic gambit approach evolutionary dynamics can be incorporated into individual based models, and that all that is required is an understanding of the probability of offspring inheriting the parental phenotype.

## Introduction

The world is experiencing unprecedented rates of environmental change [1] and it would be useful to have the ability to predict accurately the impact of such changes on the natural world. Indeed the ability to predict how a system will respond to perturbations (either experimental or natural) is a key feature of any scientific discipline [2]. Developing the ability to project the future states of ecosystems (or components of ecosystems) has not been a significant endeavour within ecology [3]. There is a considerable gap between what we currently know and what we need to know to predict, and to mitigate against, the impact of environmental change on ecological systems [1,4,5]. To make predictions about the ecological impact of environmental change ecologists require models that can be projected into future changed conditions.

Potentially, organisms can respond to changes in their environment via one or both of two routes – phenotypic plasticity and evolution. If a model is realistically to reflect an ecological response to environmental change it will need to include evolutionary change and/or phenotypic plasticity as potential responses to changed conditions [5]. Agent based models (also known as Individual Based Models - IBMs) link individuals with populations and through fitness-maximizing decisions of individuals predict population-level outcomes [6]. Thus, IBMs following phenotypes within a population [7] can be a powerful tool to predict the effect of natural selection based upon performance traits [8].

There is no consensus as to how to quantify evolutionary change and fitness in ecological time scales but there are some suggested approaches [9,10]. An as yet unutilized valuable insight comes from the ‘Formal Darwinism Project’ [11]. This project has shown by means of mathematical proof that individuals act as maximizing agents, with the proposed maximand being relative lifetime reproductive success [12]. This suggests a way in which evolutionary change can be incorporated into models at the level of the individual because, if correct, it suggests that it is reasonable to ignore the underlying genetics of traits and treat evolution as a process of phenotypic change.

Seasonally breeding organisms attempt to time their breeding such that the period of maximum food demand coincides with maximum food availability. Selection will tend to favour those individuals whose offspring have peak food demand when food is most available [13]. This raises the potential problem that if the timing of food availability changes (because climate change alters the temperature of the environment) then timing of breeding would need to change to ensure the match between food demand and food availability was retained. One of the phenomena that appears to be particularly sensitive to climate change is breeding season phenology. Some of the best examples of this are found in birds (e.g. [14]) although there are examples in many other taxa [15,16]. For example, in a survey of 65 British bird species about a third were found to be laying significantly earlier [17,18], though other studies revealed that not all populations had advanced their breeding phenology [19]. Adapting fecundity to arrival date and food availability is reported among migratory birds [20,21]. However, when the environment is changing rapidly there can be a mismatch between the timing of food availability and the timing of breeding which results in lower fitness [22].

**Figure 1:**
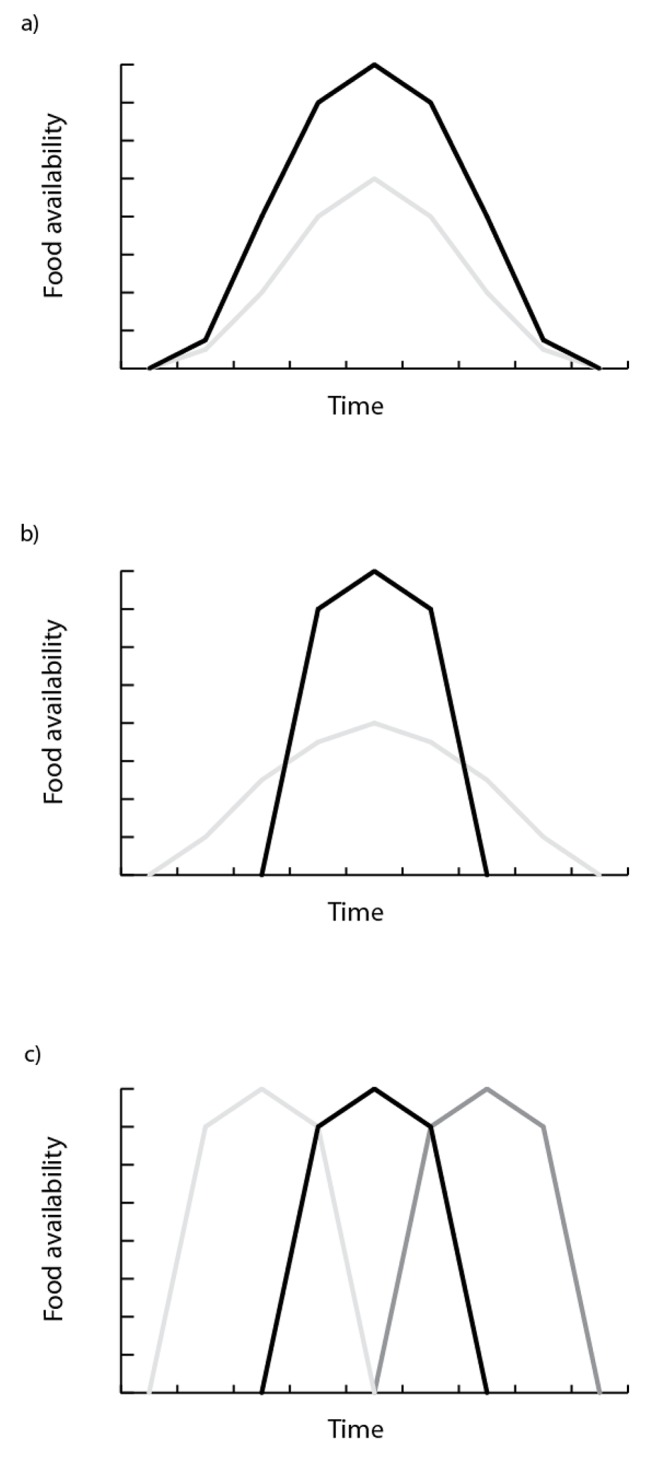
Illustration of the three variations of food availability considered in this model. a) Scenarios differ in mean food availability – dark line high mean, pale line low mean; b) Scenarios differ in the duration food availability (food availability window), dark line low variance, pale line high variance; c) Scenarios differ in the timing of food availability.

There is no doubt that timing of breeding is plastic; individuals can breed at slightly different times in different years (e.g. [23]). However, if breeding biology is substantially to change in response to changing environmental conditions (beyond phenotypic plasticity), then it needs to evolve. We considered that there could be two possible responses to changing environmental conditions during the breeding season – changing the timing of breeding and changing fecundity [19,24]. In order to examine how these traits might evolve under a range of different scenarios we established a set of simulated environments in which the absolute amount of food and the window of food availability are changed between environments while the timing of peak food availability changes between seasons within environments (Figure 1).

We have chosen to adopt the conclusions of the ‘Formal Darwinism Project’ [11] and have explored the population dynamics and fitness of phenotypes, ignoring the genetic or other mechanisms that may underlie the production of a phenotype. The principal objective of this paper is therefore to utilise this approach in order to cope with evolutionary change in an ecological model.

## Methods

The model description follows the proposed protocol for individual and agent-based models. The model is coded in C#. The code or an executable file running on Windows is available on request from the corresponding author. The model simulates seasonally breeding species that breeds just once during a season. Input parameters are not based on field data and thus the model as implemented here does not 'predict' actual phenotypic changes in a real population; the main purpose is to provide a theoretical tool for predictive eco-evolutionary modelling. We have also developed an algorithm to estimate the probability of offspring inheriting their parent’s phenotype. Here only the first three sections of the model description following protocol by [25] are listed (Purpose, State variables and scales, and Process overview and scheduling) and the rest of the model description is provided in Supp. 1. The phenotype inheritance algorithm is provided in Supp. 2.

### (a) Purpose

The model aims to follow a species that has a breeding season - the timing and fecundity of which are the traits of interest. It lives in an environment that varies spatially in the timing of food availability. Further the environment also varies stochastically year on year in the timing of peak food availability. The reproductive success of the organism is affected by the food availability during a period of time after the onset of breeding, such that individuals which experience high food availability have either more or better quality offspring. An individual’s phenotype determines the time that it breeds and its fecundity and is inherited by offspring from parents with a probability that is a function of genetic - environmental interactions (see section *(vii), g*(*ii*), and *g*(*iii*) in Supp. 1). We examine how the timing of breeding in the population responds to changes in the timing of resource availability (time of peak resources and a measure of duration of resource availability).

### (b) State Variables and Scales

All agents in the model are individuals of the same species reproducing asexually. Individuals that have not completed the breeding season in which they were produced are referred to as juveniles and individuals that have not completed a full year are referred to as immature. Individuals use resources and allocate them to growth and reproduction according to six phenotypes (*P*). The probability of an offspring having a particular phenotype given its parent phenotype is given in a probability matrix (Supp. 2), in which each probability depends on how many changes need to be made to get from parent’s to the offspring phenotype. Phenotypes stochastically predispose the timing of breeding and the number of offspring of individuals. Combinations of two phenotypic traits, one dictating the timing of breeding and one dictating fecundity, were followed [26]. The six phenotypes are referred to as: Early Prolific (EP), Early non-Prolific (EnP), Mid Prolific (MP), Mid non-Prolific (MnP), Late Prolific (LP), and Late non-Prolific (LnP) breeders. Each individual is characterized by three age variables, adult, juvenile, and immature, and the partitioning of food resources within the cell between other individuals and their juveniles through the breeding season. Survival of adults and juveniles is related to food-specific survival probability while immature survival is a random process (see section ‘Emergence’). Space is explicit in the model with a simulation grid of 30 x 30 cells. Prey availability varies between cells. Prey availability in each cell depends upon three parameters: timing of peak prey availability (*p*_*t*_), total prey availability during the breeding season (*mean*), and width of the prey availability season (*var*). All cells have (stochastically) the same mean and variance of prey availability for a given run, and these parameters are varied between runs. A high variance makes the prey distribution over the season more even, corresponding to a wider food window, but (given a constant mean of food availability) the potential maximum food quantity per week is lower although the mean amount of food available throughout the breeding season is the same. The peak prey time varies randomly (and uniformly) between years, but is fixed over each year and the same over all cells. Time follows explicitly 6 weeks of the breeding season with a time step of one week (thus six time steps each year). The rest of the time within each year is only modelled implicitly by updating the age of each surviving individual. Adults can move between each breeding season time step to any of its neighbouring 8 cells (if food is more available there), until they start breeding. After the onset of breeding adults do not move for the rest of the breeding season.

### (c) Process Overview and Scheduling

The model proceeds in weekly time steps following only six weeks per year corresponding to the breeding season. After the (six week) breeding season has been concluded, biological processes occurring outside the breading season are updated but individuals are not explicitly followed during that period. For each breeding season (six time steps): Prey availability is calculated for each week of the breeding season on each cell. Adults seek for the optimal breeding cells. Adults start breeding at time determined by their phenotype [27]. Breeding ends at the end of the breeding season (6 weeks). Adults whose phenotype does not preose them to breed during the current week keep on seeking for the optimal breeding cell until week clocking matches their phenotype clocking for breeding [28]. The number of offspring produced by each adult is determined stochastically between their phenotype (accounting stochastically for 30% of the number of offspring produced) and plasticity to the environment (accounting stochastically for 70% of the number of offspring produced) [29]. The phenotype-specific number of offspring produced by any individual is influenced by their timing of breeding phenotype, with early breeding phenotypes producing more offspring than late breeding phenotypes [21]. The phenotype-specific fecundity was set to EP = 8, EnP = 6, MP = 7, MnP = 5, LP = 6 and LnP = 4 offspring year^-1^. Early phenotypes breed during weeks 1, and 2, Mid during 3, and 4, and Late during 5, and 6. Breeding always lasts one week. Offspring phenotype is stochastically inherited from parent (Supp. 2). In the case that the offspring does not inherit the parental phenotype it acquires one of the remaining 5 phenotypes with a certain probability (Supp. 2). The offspring survival depends (stochastically) on food availability in its cell (food is partitioned between adults and juveniles) at the time of its birth and in subsequent weeks. Offspring survival is a natural selection process occurring stochastically but based upon prey availability in the cell and density dependence for partitioning food resources among adults. At the end of the six week breeding season: Adult survival is a natural selection process of individuals which is a function of number of offspring produced and total prey handling of the individual throughout the breeding season [28]. Offspring (juveniles) turn into immatures. At the end of the year: immatures mature into adults.

### Fitness

In order to provide an indicator of phenotype-specific fitness we followed the individual’s contribution to population growth *p*_*ti*_ following the method (de-lifing) proposed by [10], as the method can be implemented in overlapping generations. The method calculates lifetime reproductive success on an annual time step. The method calculates how a population would have performed with the focal individual removed over the time step *t* to *t*+1, and it is implemented by retrospectively removing the individual and any offspring that were produced between *t* to *t*+1 that are still alive at *t*+1 and recalculating population growth [10]:


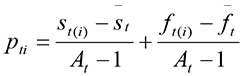


, where *s*_*t*(*i*)_ is a binary variable representing whether individual *i* survived from year *t* to *t*+1, and *f*_*t*(*i*)_ is the number of offspring produced by individual *i* in year *t* that survive to year *t*+1 and *s̄*_*t*_ and *f̄*_*t*_ are the means of *s*_*t*(*i*)_ and *f*_*t*(*i*)_, while *A*_*t*_ are adults in time step *t*. Fitness *p*_*ti*_ was calculated for each of the six phenotypes for each simulation scenario and the relative contribution of individuals of each phenotype to the total population (individuals of all six phenotypes) in the examined simulation scenario was recorded.

### Statistical Analysis

Linear mixed effects models fitted with maximum likelihood were used to assess significant relationships between individuals of each phenotype (dependent variable, repeated 6 times for each phenotype). The model structure included total prey availability (*mean*), window of prey availability (*var*), as fixed effects, peak week of prey availability during the breeding season (peak2 ..., peak6) as fixed factor, and year as a random effect. Inspection of residual plots for constancy of variance and heteroscedasticity indicated that the models were well behaved in all cases. Statistical analysis was conducted in *R* 2.12.0 [30].

### Experimental model variants

We sought to quantify whether the level of complexity in the model is justified [31]. We manipulated model properties (rules) and recorded the same parameters (see section 'Observation'). Outputs of different model complexity levels were compared with the 'normal' model case described in this paper in order to examine the level of complexity that is justified. Note that essentially this exercise should be conducted by comparing model outputs deriving from different levels of model detail & complexity with data; This model is not calibrated with field data and we are thus unable to compare model outputs with data, we are comparing model outputs with model outputs deriving from different complexity levels as an example of how such comparisons are feasible. To this end we have created variants of the model in which: (a) There is no timing - all individuals breed in the middle of the breeding season (Only Mid phenotypes MP and MnP with phenotype-specific offspring of 7 and 5 per year respectively). (b) There is no space - the model has no spatial component, i.e. the environment consists of a single cell. (c) There are no phenotype transitions (i.e. all offspring inherits parent’s phenotype) (d) There is no time-specific productivity- all prolific phenotypes produce 7 while all non-prolific phenotypes produce 5 phenotype-specific offspring per year regardless of the time of breeding within the breeding season). Outputs for the different model complexity levels were compared against the first (mean=15, var=3) and the last (mean=35, var=18) prey availability simulation scenarios.

## Results

**Figure 2:**
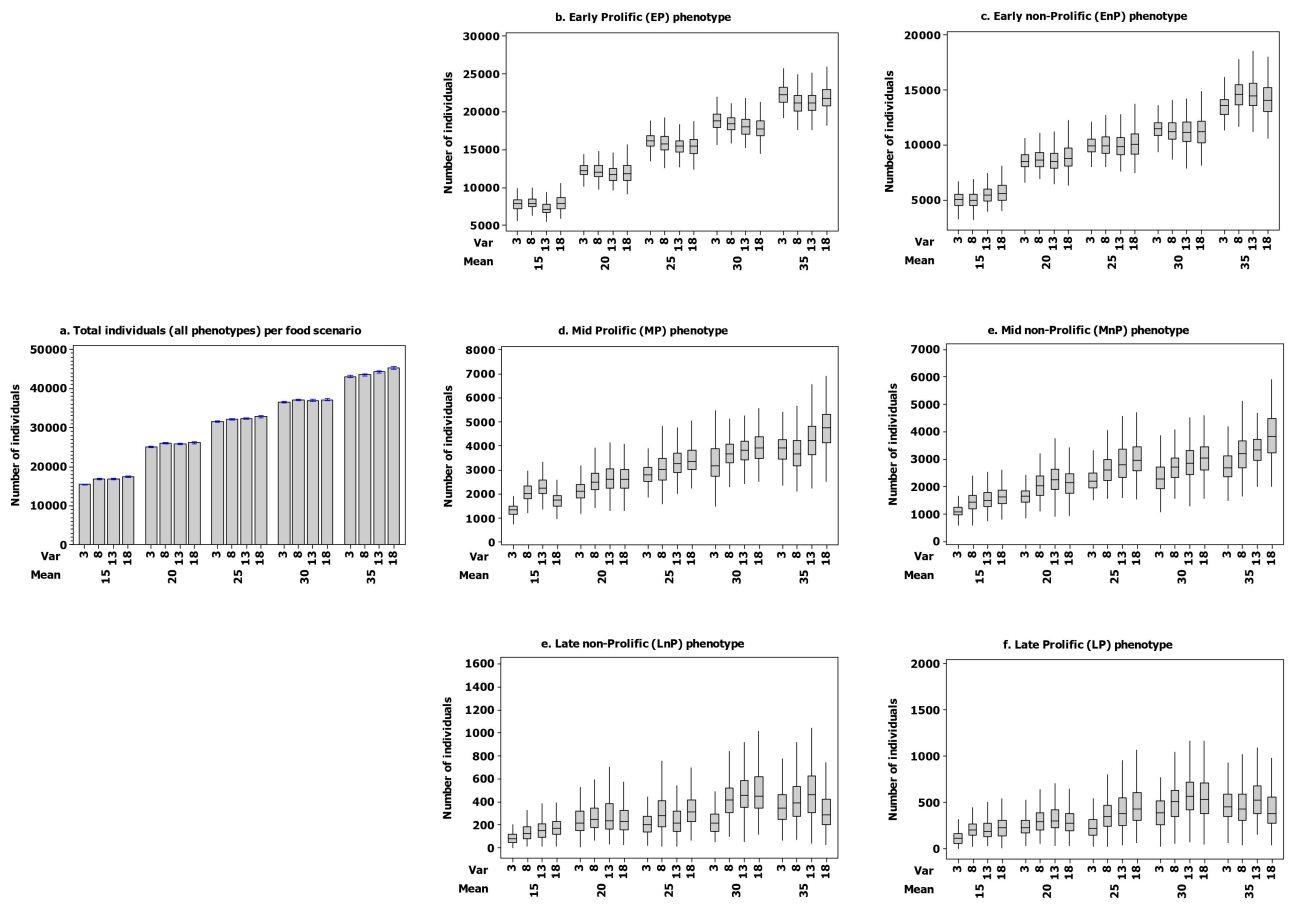
a. Total mean number of individuals (summing all 6 phenotypes) per simulation scenario across 300 simulation years. (b-g) Boxplots of (b) Early Prolific, (c) Early non-Prolific, (d) Mid Prolific, (e) Mid non-Prolific, (f) Late Prolific, and (g) Late non-Prolific phenotypes across all simulation scenarios. Values in vertical axis are individuals per phenotype, horizontal axis depicts total food availability followed by window of food availability per simulation (see 'Initialization' for a full description of simulation scenarios). Horizontal lines within boxplots depict mean values, the top of the box is the third quartile Q3 (75% of the data values are less than or equal to this value) the bottom of the box is the first quartile Q1 (B25% of the data values). The upper whisker extends to the highest data value within Q3 + 1.5 (Q3 - Q1). The lower whisker extends to the lowest value within Q1 -1.5 (Q3 - Q1).

In environments in which there was a high food availability there were high total number of individuals in the population, while increasing the duration over which food was availability marginally increased the total number of individuals (Figure 2a). The increase in the number of individuals with food availability was not consistent in all phenotypes; early breeding phenotypes and, to a lesser extent, prolific phenotypes were more sensitive to changes in food availability than others (Figure 2b–2g, Supp. 3).

**Figure 3:**
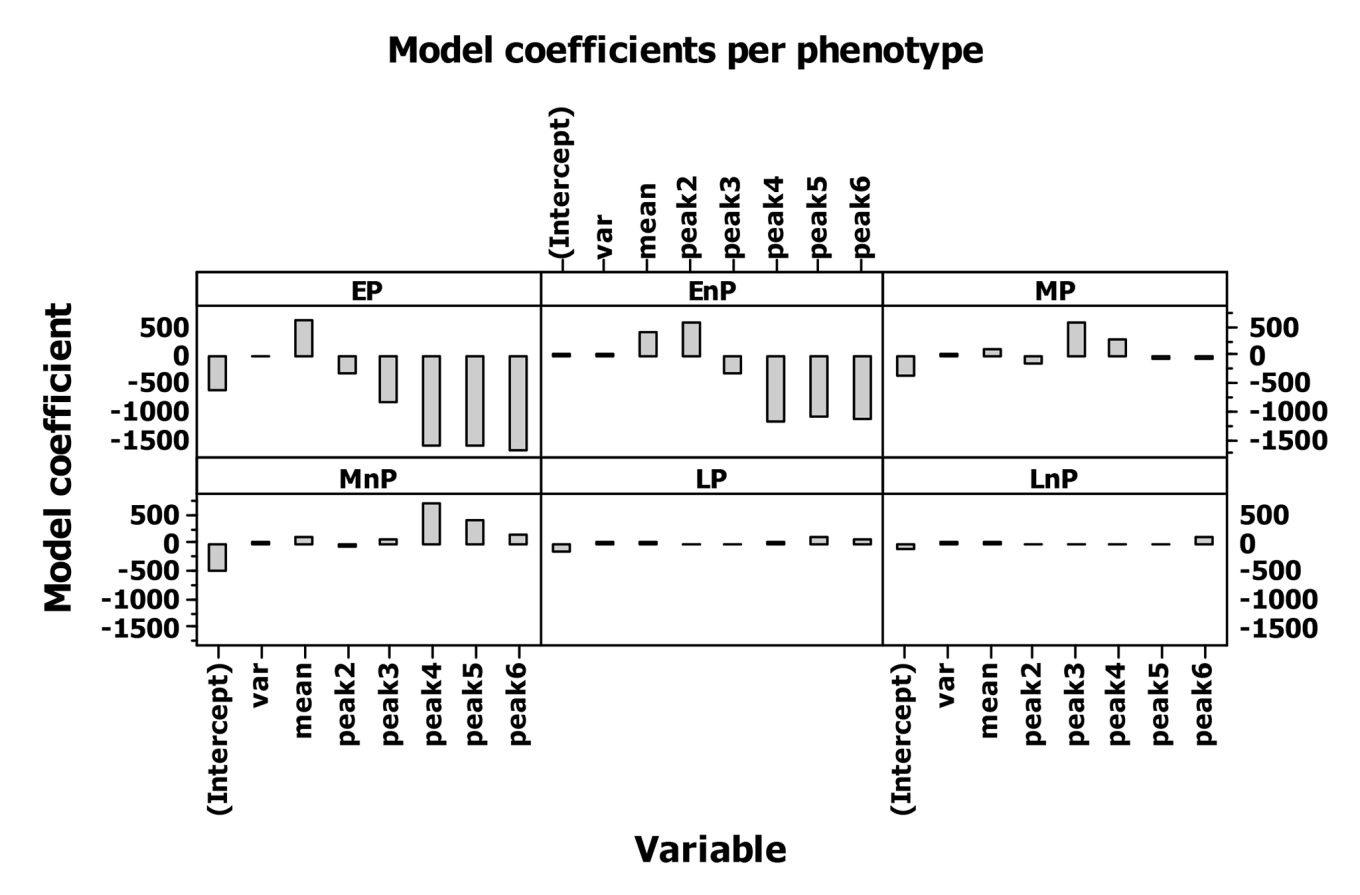
Coefficients of mixed effects models per phenotype. Coefficients of linear mixed effects models of individuals per phenotype (dependent variable). The model structure included total prey availability (*mean*), window of prey availability (*var*), as fixed effects, peak week of prey availability during the breeding season (peak2 ..., peak6) as fixed factor, and year as a random effect. For details regarding the statistical analysis see section 'Statistical analysis' and Supplement 3.

The phenotype-specific linear mixed effects models indicate that for EP phenotypes increasing the window of food availability (*var*) had a negative effect on population sizes, while increasing food availability had a large positive effect. The maximum population of EP phenotypes was attained when food availability peaked in week one and any departure of early peak in food had a large negative effect (Figure 2 and Figure 3; for details see also Figure 1 in Supp. 3). The population of EnP phenotype also decreased with large windows of food availability, and increased with increased food availability, for this phenotype maximum population size was reached when food availability peaked early but not the earliest possible (maximum attained with a week 2 peak); (Table 1; Figure 2 and Figure 3).

**Table 1:** Summary of effects of total food availability, length of the window of food availability and the timing of peak food availability on population sizes of different phenotypes.

	EP	EnP	MP	MnP	LP	LnP
Total food availability	+	+	+	+	+	+
Length of the window of food availability	-	+	+	+	+	+
Week in which peak of food availability most positive	1	2	3	4	5	6
Week in which peak of food availability most negative	4,5,6	4,5,6	1	1	1	1

The population of MP phenotype decreased with large windows of food availability, and increased with increased food availability. The optimal peak timing of food for this phenotype was week 3 (early mid season) followed by week 4 (late mid season). Early peak (weeks 1 & 2) had a negative effect on the population size of the MP phenotype with a less strong negative effect of a late peak (weeks 5, & 6); (Figure 2 and Figure 3). Larger windows of food availability as well as high total food availability had a positive effect on the population sizes of the MnP phenotype. Populations of the MnP phenotype were higher when the peak of food availability was fairly late in the season (week 4) but any late peak (week 5 & 6) was positive.

Large windows of food availability as well as high total food availability had a positive effect on population sizes of the LP phenotype. For this phenotype the optimal timing of the peak of food availability was towards the end of the breeding season (week 5), but any mid–late (week 4) or very late (week 6) peak had a positive effect (Figure 2 and Figure 3). Similarly, the population sizes of the LnP phenotype were higher when the window of food availability was large as well as when there was high total food availability. The optimal timing of the food peak was during the last week of the breeding season (week 6) for this phenotype. An early peak (week 2) had an almost negligible effect on the population size of LnP while a mid or late peak apart from very late (weeks 3, 4, and even 5) has a negative effect (Table 1, Figure 2, and Figure 3).

In all phenotypes the effect of total food availability on population size was more pronounced than the effect of width of the window of food availability. The effect of total food availability on population size was more pronounced in early phenotypes than in late phenotypes, and more pronounced in prolific than in non-prolific phenotypes (Figure 2 and Figure 3). These effects are summarized in Table 1. Detailed results of mixed effects models are provided in Supp. 3.

**Figure 4:**
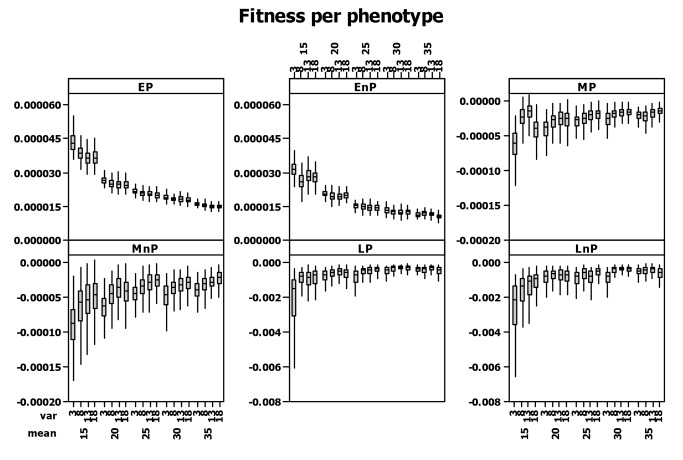
Fitness per phenotype calculated as the individual’s contribution to population growth on an annual basis following the method (de-lifing) proposed by Coulson et al. (2006a). The method calculates how a population would have performed with the focal individual removed over the time step *t* to *t*+1, and it is implemented by retrospectively removing the individual and any offspring that produced between *t* to *t*+1 that are still alive at *t*+1 and recalculating population growth (see section 'Fitness').

The relative contribution of individuals to the population was much greater for early phenotypes than for mid and late phenotypes (Figure 4). Within that pattern prolific phenotypes always contributed relatively more to the overall population fitness than non-prolific phenotypes. Both mid and late phenotypes typically had negative fitness; early phenotypes made a greater contribution to the overall population fitness at low food availability scenarios than at high food availability. Although mid and late breeding phenotypes still had negative fitness at high food availability it was relatively higher than when food availability was low. Overall, short windows of food availability (low values of *var*) were associated with higher fitness differences between individuals of the same phenotype.

**Figure 5:**
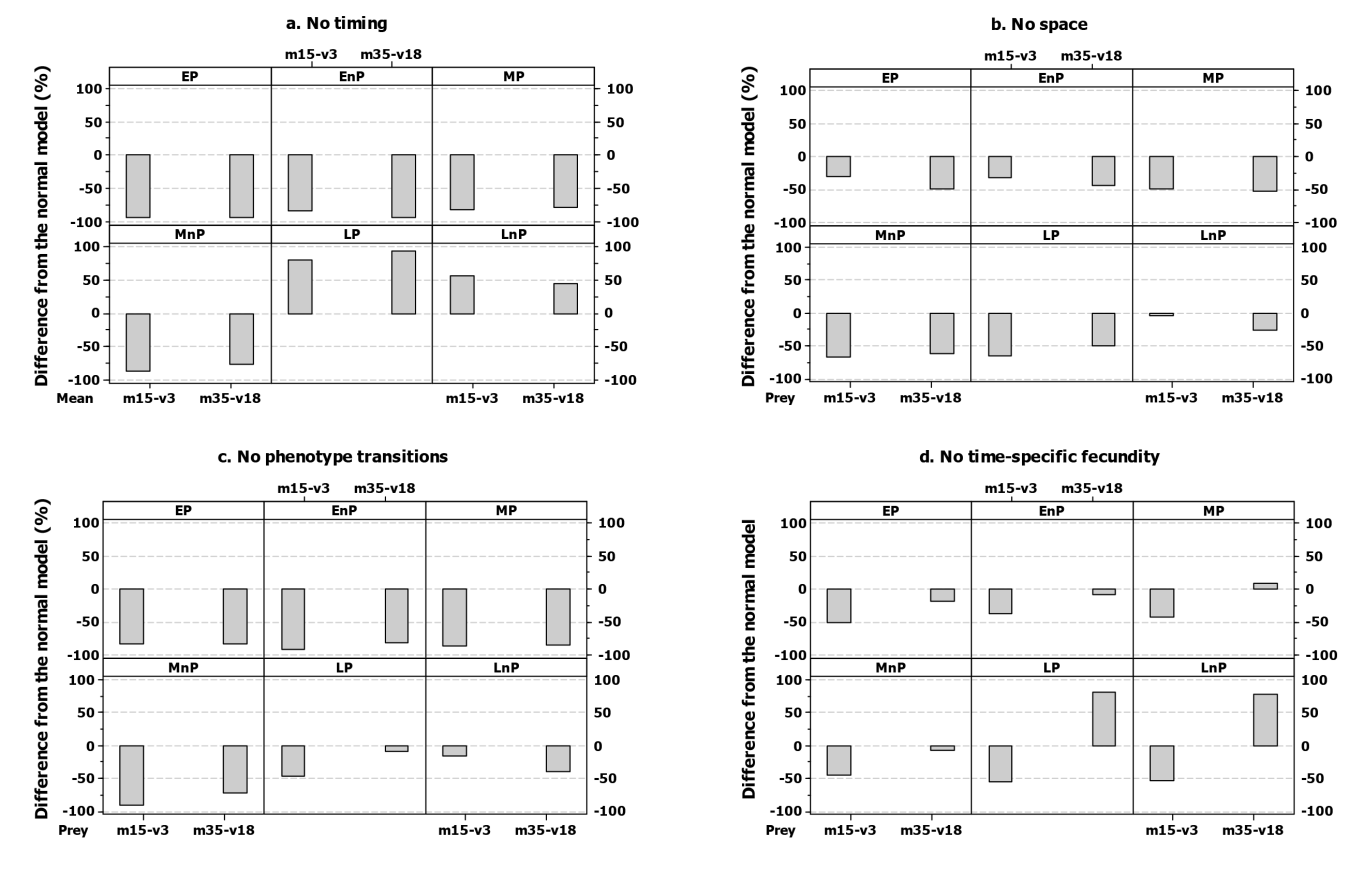
Hypothetical replication of the first (a. mean=15, var=3) and the last (b. mean=35, var=18) prey availability simulation scenarios with different levels of model complexity: (a) No timing - only Mid phenotypes MP and MnP with gene-specific offspring per year 7 and 5 respectively. (b) No space - the environment consist of a single cell. (c) No phenotype transitions (all offspring inherits parent’s phenotype). (d) No time-specific productivity- all prolific phenotypes produce 7 while all non-prolific phenotypes produce 5 phenotype-specific offspring per year. Outputs for the different model complexity levels were normalised against the values of each phenotype in the 'normal' model output. Values are given as a % of the difference between: [(simpler model - normal model) / normal model] outputs per phenotype.

Any simpler model than the one used here produced substantial changes in the populations of all phenotypes (Figure 5). When no timing in the model structure is included late phenotypes are overestimated while mid and early phenotypes are severely underestimated (Figure 5a). Outputs from the spatially implicit model underestimated the number of individuals across all phenotypes (Figure 5b). When no phenotype transitions are included (i.e. offspring always inherit their parent’s phenotype) numbers of all individuals are always underestimated but this was more pronounced in early and mid breeding phenotypes (Figure 5c). A model that did not include a time-specific differentiation in the number of offspring produced per phenotype underestimated early and mid phenotypes, particularly when prey availability was low and of short duration (Figure 5d) while it overestimated late phenotypes, in particular when prey availability was high and of long duration (Figure 5d). The relatively high success of early breeding individuals is not simply the result of the fact that they produce more offspring than late breeding individuals as these still remain the most successful phenotype when the fecundity differences between birds breeding at different times are removed.

## Discussion

Predicting the likely impacts of environmental changes is becoming increasingly important, as we try to identify and describe sets of plausible futures given different scenarios of change [32,33]. It is difficult to envisage how such forecasts could be produced using anything other than computer models and that, because of the need to project them into novel future conditions, such models would need to be process-based rather than phenomenological [1,4]. While for long-lived organisms (relative to the length of the model run) it may be possible to ignore evolutionary change (as is done for trees in forest gap models [34,35], in most cases it will be desirable to include the capacity for adaptation through either phenotypic plasticity or evolution. This seems to be done only rarely [5].

Incorporating evolution within ecological modelling has long been recognized as a challenging task. Initial attempts were based on differential equations seeking analytically optimal mathematical solutions [36–39]. These approaches aimed at providing (through analytic calculations) the solution to genetic models but they were neither calibrated with data nor tested against existing data. More recent integral projection models (IPM) have been proposed for integrating eco-evolutionary dynamics and tested against data (e.g. [40]). Further, rigorous methods for disentangling genetic - environmental interactions have been proposed [41] and were also tested against data [8]. The approach that we have taken here is to use a computational approach, which is spatially explicit and allows population dynamics to emerge. While applying these approaches are not mutually exclusive with the approach proposed here, we argue that the approach proposed here is simple (all that is required is quantifying the ratio of phenotype inheritance from parents to offspring) but, most importantly, such a model can demonstrate eco-evolutionary dynamics.

There have been some attempts to incorporate evolution into ecological modeling computationally: one attempt has been to create a thought experiment - imagine an ecosystem and generate insights about what might happen if the world were to be organised as described [7,42,43]. These results are not compared to data from a real-life system, nor is there any claim that the model represents any specific system or species. Nevertheless we can generate insights about what might happen if the world were to be organised as imagined in such a manner. Another way forward is to create an IBM that includes the capacity for evolutionary change using ‘genetic’ rules and allow the behaviour of individuals to emerge from changes in the genes that code for the behaviour of interest, and this route was taken by [44] though the model was not explicitly calibrated with data at the level of the gene. Finally, an IBM can be calibrated with data at the level of gene and explicitly account for both ecological and evolutionary dynamics [45]. However, this is highly demanding of data and may be a complication too far [46]. Most environmental stakeholders are not particularly interested in changes in gene frequency; what is observed and the level of organization about which people are typically concerned is that of the individual organism. In addition, computing the solution to a genetic model may not always be particularly useful; there will be a wide range of potential models that differ in the details of the genetic mechanisms that underlie them (and on which we are unlikely to have data). In the model presented here we have chosen to utilize differences in phenotype. We argue that this is potentially both more useful and more relevant to the types of question that typically need to be addressed. In taking this stance we are essentially adopting the arguments made for ignoring the underlying genetics in ESS models – the so called ‘phenotypic gambit’ [47]. In his ‘Formal Darwinism Project’ Grafen has provided robust arguments for the use of the individual as maximizing agent, with the proposed maximand being relative lifetime reproductive success [12]. He has subsequently demonstrated that this approach is equivalent to the alternative that may be obtained by a population genetic analysis of changes in genotype frequencies [11]. We propose that a combination of ‘Formal Darwinism Project’, providing a justification for examining a system at the level of phenotype rather than genotype, together with 'de-lifing' [9,10], which provides a robust fitness index at the level of individuals may be used as a link between ecology and evolution. Our conclusion regarding the principal objective of this work is that the phenotypic approach has utility when attempting to accommodate evolution within an ecological model. The main issue that is resolved is that there is no need to make assumptions about the underlying genetics of the system; all that is required is an understanding of the probability of offspring developing a different phenotype from their parent. Such data are fairly easy to collect.

In order to illustrate this approach we have explored the relatively simple problem of when and at what level individuals organism should breed using an agent based model using as an example a seasonal breeding species and focusing on differences between phenotypes and their relative contribution to the overall population fitness. We have shown that the frequency distribution of different phenotypes changes in response to changes in the timing and scale of food availability during the breeding season. Starting with equal numbers of the six phenotypes at the start of each simulation we rapidly move to a situation where one phenotype predominates. Despite the fact that one phenotype – early and prolific breeding – tends to dominate the population we never see complete extinction of any of the six phenotypes. This is partly due to the fact that phenotypes are constantly regenerated by transitions to other phenotypes but also because there are some circumstances in which these alternatives phenotypes perform well. The observation that all phenotypes continue to exist in the scenario that has all offspring inheriting their parental phenotype shows that their persistence is not simply due to them being regenerated through transitions. Overall, as implemented in the model, adult survival, offspring survival as well as the number of offspring produced (optimal clutch size), factors that ultimately determine survival and fitness, are implemented in the model by genetic - environmental interactions: Adults breeding late and immatures born late in the season have the advantage that they are less exposed to food shortage late in the season, as adults can reduce the number of immatures that they produce based upon (low) food availability (p 122 in [48]). Adults breeding late and immatures born late have the disadvantage that there is a higher crowding effect late in the season as there is a higher number of adults and immatures partitioning food resources (p 122 in [48]). Further, the model accounts for the lifetime reproductive success as individuals adapt the number of offspring that they produce each breeding season (they may even not breed) upon food availability, modulated by the environmental factor in the genetic-environmental interaction. It is important to note that this is in the absence of a directional signal in the timing of food availability. If such a signal were to be imposed we would anticipate that timing breeding would respond especially if this signal was such that favoured early breeding.

How simple can the model become before it loses its ability to generate realistic predictions? In the theoretical exercise conducted here, the importance of retaining complexity is shown by the large change in the numbers of each phenotype that were obtained when we forced all offspring to inherit the parental phenotype. It should be noted however that the result that simpler models somehow under or over-estimated various phenomena when their details were removed derives here by comparing model outputs with each other and not model outputs with data, as it would be preferable. Sometimes simpler models may be better calibrated to reality and a more detailed model can over or under estimate reality - it is more a precise perhaps but less accurate representation of reality. Ultimately this exercise should be repeated by comparing model outputs with data and this work here serves as an example of how this is feasible. It is going to be rare that we have a robust understanding of genetic mechanisms for any trait of ecological interest, but data on the probability of offspring inheriting the parental phenotype are not as difficult to collect. Allowing offspring to develop phenotypes other than that of their parent probably results in higher overall populations because in suboptimal conditions for your phenotype [49], offspring of non-parental phenotypes will perform better than offspring of the parental phenotype. Allowing offspring to develop phenotypes other than that of their parent therefore reduces the number of ‘eggs in one basket’, similar to an ‘tangled bank’ argument for the evolution of sex [50] and could be regarded as comparable to one of the classic questions in ecology 'Does diversity beget stability?' [51]. We also conclude that the spatial dimension is important; the real world is spatially explicit – individuals are more likely to interact with neighbours than individuals a long way away and to move to nearby locations rather than distant ones. If we do not include spatial structure in our model the numbers of all phenotypes falls but importantly does not fall in a uniform manner, e.g. populations of late breeding non-prolific individuals are barely affected while early prolifically breeding phenotypes are approximately halved. So making the model spatially implicit changes the results both qualitatively and quantitatively, as has been found in other studies [31]. In our view the changes that were seen when the full model was simplified justifies the use of the current level of complexity.

## Supporting Information

Figure S1 Detailed results of linear mixed effects models of individuals per phenotype (dependent variable).
(TIF)

Supplement S1 Full model description.
(DOC)

Supplement S2 Phenotype inheritance probabilities.
(DOC)

Supplement S3 Detailed results of linear mixed effects models per phenotype.
(DOC)
