# Supplementary figures and images for: Integrating Evolution into Ecological Modelling: Accommodating Phenotypic Changes in Agent Based Models
Source: PLoS One. 2013 Aug 5;8(8):e71125. doi: 10.1371/journal.pone.0071125 (PMC3733718; doi:10.1371/journal.pone.0071125)

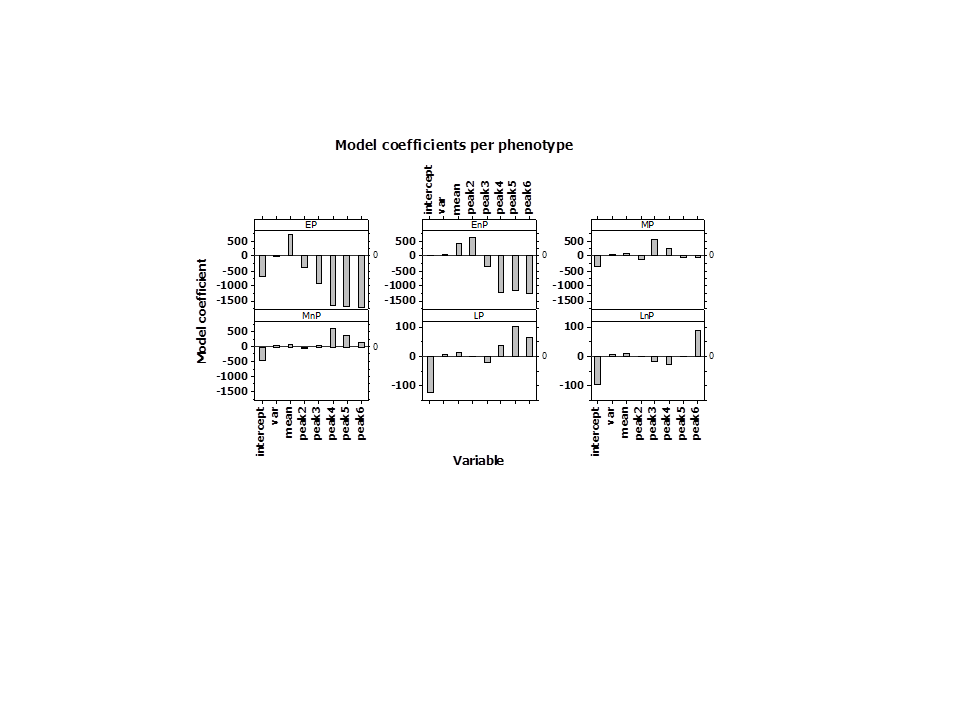

Supplement: Figure S1 — Detailed results of linear mixed effects models of individuals per phenotype (dependent variable). (TIF) [file pone.0071125.s001.tif]
