# Supplementary material for: Integrating Evolution into Ecological Modelling: Accommodating Phenotypic Changes in Agent Based Models
Source: PLoS One. 2013 Aug 5;8(8):e71125. doi: 10.1371/journal.pone.0071125 (PMC3733718; doi:10.1371/journal.pone.0071125)
Supplement: Supplement S1 — Full model description. (DOC) [file pone.0071125.s002.doc]

**Supplement 1. Full model description**

*(d) Design concepts*

*(i) Emergence*

Population life histories emerge from environmental stochasticity regarding temporal and spatial prey availability, parent’s investment in growth, fecundity, and reserves. Individual’s decisions are always stochastic and involve either genetically programmed decisions or involve combinations of genetic and environmental interactions in terms of plasticity to prey availability . Density-dependence occurs for partitioning of food resources only, which in turn affects population demography . Individual’s survival and phenotypic propagation through natural selection depends upon individual rules such as fecundity and timing of reproduction, and environmental stochasticity through total prey availability, timing of prey availability and competition for prey resources partitioning. There are three types of mortalities leading to natural selection. (i) Adult mortality is determined by the partitioning of the total number of prey available in the cell throughout the breeding season between the number of adults in the cell and the number of offspring produced by the adult . The values used here were set to 40% and 60% respectively. (ii) Juvenile mortality is dependent upon food intake from parents on each week of the breeding season . The value used here was (arbitrarily) set to minimum = 242 g week^-1^. (iii) Immature mortality occurring through a random death process as a percentage of the immature population corresponding to selection processes non-related to food intake such as predation or weather conditions . Immature annual mortality ratio was set to 30%.

*(ii) Sensing*

Individuals are assumed to know their phenotype determining the onset of breeding timing and the number of offspring produced. Individuals sense population and prey availability in current and 8 adjacent cells.

*(iii) Interaction*

Individuals do not mate nor interact directly; individuals interact indirectly through competing for prey resources. Individuals start a year on the cell it was on at the end of the last breeding season and then at the end of each time step until it starts breeding it can move to the 8 adjacent cells. The searching criterion that they try to maximize by moving is to locate the cell with the highest food availability per capita (prey resources in the cell / individuals breeding in the cell). Individuals stop moving after the onset of breeding. Individuals are not explicitly followed during the rest of the year (non-breeding season). During the successive year individuals are located into the cell in which they bred last year and start searching among neighbouring cells for the optimal site for breeding. Movement is continued on each time step until the onset of breeding. Note that individuals can move to one of the neighbouring 9 cells between each time step and not throughout the simulation i.e. during the successive time steps the 9 cell neighbourhood is not necessarily the same as the one during the previous time step.

*(iv) Stochasticity*

Stochasticity occurs in timing and amount of prey throughout the breeding season, prey spatial distribution, adult, nestling and immature survival, and offspring phenotype inheritance. The peak week of prey availability period is stochastic. Prey availability is also a stochastic Gaussian variable around a mean and standard deviation influencing the maximum prey availability value for the breeding season and the width of the prey availability window through the breeding season. Given a constant mean number of food availability, high variance will tend to give a more even food supply during each week (as the Gaussian function shape is wider with higher variance) but during the peak week of food availability, the food available will be lower than in lower variance scenarios. This is because each breeding season 6 numbers from a Gaussian distribution with parameters defined by *mean and var* are drawn and sorted so as to peak during week peak week.(see also section submodels: *(vii) Prey Availability*).

After the onset of breeding the number of offspring per adult is a genetic x environmental interaction modulated by stochasticity. The phenotype of each offspring is inherited by parent adult with a certain probability. Mortalities of adults and juveniles are stochastically modulated by food intake and immature mortalities are stochastic around a mean annual death rate.

*(v) Observation*

All variables are stored on an annual basis. The recorded variables are: the three parameters of prey availability, the age and phenotype of each adult (each with a unique ID number), the number of offspring of each adult and their phenotype, and the number of deaths of adults, juveniles, and immatures and their phenotypes.

*(e) Initialization*

Individuals are initially randomly distributed in the grid. Initial peak value of prey availability period is also randomly chosen. Equal number of all phenotypes was initially set. Simulations were run for 320 years discarding the first 20 years. In total 5 total prey availability scenarios (*mean* = 15, 20, 25, 30, 35) were examined, each replicated by 4 prey availability season windows (*var* = 3, 8, 13, 18) resulting in 20 simulation scenarios in total. Prey availability values are referred to the total amount of prey available per cell per breeding season. Peak prey availability week is chosen randomly for each year, with weeks 1 through 6 being equally likely, and this is the same for all cells.

*(f) Input*

The gene-specific number of offspring produced on average during each breeding season (Supp. 2) and the phenotype-specific timing (week) of the onset of breeding (see 'Onset of breeding') are read from file. Total prey availability and width of prey availability during the breeding season are input parameters (see 'Initialization').

*(g) Submodels*

*(i) Onset of breeding*

Adults have a phenotype-specific time (week) on which they start breeding during the breeding season. Note that the onset of breeding is independent from prey availability. Early breeders start breeding during week 1 or 2 of the breeding season, mid breeders start breeding during week 3 or 4 of the breeding season, and late breeders start breeding during week 5 or 6 of the breeding season by drawing a random number in [1, 2], [3, 4], and [5, 6] respectively. Breeding allways lasts one week.

*(ii) Number of offspring per adult per breeding season (O_i_)*

The number of offspring produced per adult depends on the its phenotype and on the amount of prey available on current cell during the week of the onset of breeding. The week of the onset of breeding depends on the individual's phenotype.

$$O_{i}=(c_{1}P_{i}+c_{2}\frac{F_{ijt}}{A_{ijt}})*c_{o}^{-1}$$

where *O_i_* is the number of offspring (juvenile) per individual *i*, *c_1_* ( = 0.3) and *c_2_* ( = 0.7) are constants, *P_i_* is the mean number of offspring produced by individuals of phenotype *i* per breeding season, *F_ijt_* is the prey availability in cell *i*, *j*, during time step (week) *t*, and *A_ijt_* is the number of adults in cell *i*, *j*, during time step *t.*. $c_{o}^{-1}$(= 1) is a correction factor (not used). *P_i_* values are listed in Table 1, Supp. 2.

*(iii) Determining the phenotype of each offspring*

Adults have a phenotype-specific inheritance probability *w_i_* of their phenotype *i* to their offspring (Supp.1). Each line *i* (*w_i1_ + w_i2_ +…+w_i6_*) in Supp. 2 adds to 100. For each offspring produced by an adult a random number (*rnd*) in (0, 100) is drawn and the nearest value *w_iz_*. smaller than *rnd* determines the phenotype of the offspring by setting the phenotype into phenotype *z*. For example if parent's phenotype is EP and *rnd* = 51 then from Supp. 2, Table 1, line 1, we see that the nearest smaller value is *w_11_* = 46 and thus the phenotype of this offspring is set to EP. This process is repeated for as many times as the number of offspring produced by the focal individual during current breeding season.

*(iv) Juvenile survival (S_N_)*

Juvenile survival is calculated with the assumption that food is divided among all adults in a cell at each time step and that adults feeding offspring need also to find food for their offspring which is not consumed by themselves, and sum the food to adult and their offspring ratio to get its portion. Then that portion is multiplied by a correction factor *c_N_* that converts this ratio to survival:

$S_{N_{i}}=\frac{F_{ij}}{(c_{3}A_{ij}+c_{4}N_{ij})*c_{N}}$

where *S_Ni_* is the probability of survival of juvenile *i*, *c_3_*, *c_4_* (c_3_ = c_4_ = 0.5) are constants, *F_ij_* is prey availability in cell *i*, *j*, *A_ij_* is the number of adults in cell *i*, *j*, and *N_ij_* is the total number of juveniles in cell *i*, *j*. Juvenile survival is calculated for every juvenile (after a juvenile is born) during every week during the breeding season.

Overall, following the above formula, immature mortality is dominated by low food periods after birth. This gives immatures born late in the season the advantage that they face mortality over fewer weeks and the disadvantage that there is a higher crowding effect late in the season as there is a higher number of immatures in the cell.

If, for example, there are (mean) 35 units of food available per week and ≈48 adults in the cell at the end (total population ≈43000 see Fig. 2a, in 30 x 30 = 900 cells), and each adult has produced 5 offspring then the formula gives an offspring survival probability per week of 35/[(0.5*48+0.5*48*5)*0.004] = 60.75

*(v) Immature survival (S_F_)*

Immature death rate at the end of each breeding season is value *S_F_* ( = 30%) that varies randomly ± 5% each year using a uniform random number generator. Immature survival is calculated once per year after the termination of the breeding season.

*(vi) Adult survival (S_Ai_)*

Adult survival is calculated with the assumption that food is divided among all adults in a cell at each time step and that adults feeding offspring need also to find food for their offspring which is not consumed by themselves, and sum the food to adult and their offspring ratio to get its portion. Adult survival is calculated upon the total amount of food available throughout the breeding season (6 weeks) and not on a per-week basis because adults can survive s a period of time with lower food resources; However, surviving through the winter depends upon the total amount of food that the individual had access to during the season. Then that portion is multiplied by a correction factor *c_a_* that converts this ratio to survival.

$$S_{Ai}=\frac{\sum_{t=1}^{t=6} F_{ijt}}{(c_{5}A_{ijt}+c_{6}O_{i})*c_{a}}$$

where *S_Ai_* is the probability of survival of adult *i*, $F_{ijt}$ is the prey that is available in cell *i*, *j*, at time step *t* and $\sum_{t=1}^{t=6} F_{ijt}$ is the total prey available to the adult *i* throughout the breeding season. Note that adults may move between cells prior to breeding and thus breeding cell may not be the same as a cell that the adult was prior to the onset of breeding but during the breeding season and that is tracked in each individual). *A_ijt_* is the number of adults in cell *i*, *j*, at time step *t*, *O_i_* is the number of offspring (juveniles) that the adult *i* has produced during the breeding season, and *c_5_* ( = 0.4), and *c_6_* ( = 0.6), *c_a_* (= 11) are constants.

If for example there are 210 units of food total available over the season (mean of 35 times 6 weeks) and ≈48 adults in the cell at the end (total population ≈43000 see Fig. 2a in 30 x 30 = 900 cells), and the focal adult has produced 5 offspring then the formula gives an adult survival probability per year of 210/[(0.4*48+0.6*5)*6] = 0.86.

*(vii) Prey Availability*

Prey availability every breeding season is calculated based upon three parameters: (i) peak week of prey availability (*p_t_*) that defines which week prey availability will be maximized, (ii) the total value of prey to be available per cell during the breeding season (*mean*), and (iii) the width of prey availability window - variance (*var*). Peak week of prey availability is calculated by drawing a random number in [1,…, 6]. *mean* and *var* are constant parameters throughout a simulation run and are read from a file. Sequentially 6 numbers from a Gaussian distribution with parameters defined by *mean and var* are drawn and sequentially sorted so as to peak during week *p_t_*. This process defines prey timing and availability for each cell of the simulation grid during each breeding season. Note that this process is repeated independently for every cell. Thus all cells have the same Gaussian mean and variance of prey availability throughout all years, prey peak week is random between years but identical for all cells within a year, but within each week of the breeding season, prey availability varies stochastically between cells. This is because 6 numbers of a Gaussian distribution around the constant mean and variance are drawn and sorted around the peak week for each cell (900) and thus prey availability differs between cells but remains synchronized with respect to the time of peak prey.

**References**

1. Benton TG (2012) Individual variation and dynamics: lessons from a simple system. Philosophical Transactions Of The Royal Society Of London Series B-Biological Sciences 367: 200-210.

2. Dijkstra C, Daan S, Buker JB (1990) Adaptive Seasonal-Variation In The Sex-Ratio Of Kestrel Broods. Functional Ecology 4: 143-147.

3. Martin TE (1987) Food As A Limit On Breeding Birds - A Life-History Perspective. Annual Review Of Ecology And Systematics 18: 453-487.

4. Sullivan KA (1989) Predation and Starvation: Age-Specific Mortality in Juvenile Juncos (Junco phaenotus). Journal of Animal Ecology 58: 275-286.

5. Pugesek BH, Diem KL (2008) The timing and location of mortality of fledgling, sub-adult, and adult Callifornia Gulls. The Wilson Journal of Ornithology 120: 159-166.
