# Supplementary material for: Integrating Evolution into Ecological Modelling: Accommodating Phenotypic Changes in Agent Based Models
Source: PLoS One. 2013 Aug 5;8(8):e71125. doi: 10.1371/journal.pone.0071125 (PMC3733718; doi:10.1371/journal.pone.0071125)
Supplement: Supplement S2 — Phenotype inheritance probabilities. (DOC) [file pone.0071125.s003.doc]

**Supplement 2**

*Phenotype inheritance probabilities*

The model follows two traits (phenotypes) one that influences timing of breeding (Early, Mid, Late) and the other the number of offspring per breeding season (Prolific and non-Prolific). Combinations of two phenotypes result in six phenotypes in total: Early Prolific (*EP*), Early non-Prolific (*EnP*), Mid Prolific (*MP*), Mid non-Prolific (*MnP*), Late Prolific (*LP*), and Late non-Prolific (*LnP*) breeders. An offspring's phenotype is inherited from its parent with probability *u* (note this applies to one of the two traits – see below). In case the offspring does not inherit the parental phenotype it acquires one of the remaining 5 phenotypes with a certain probability. We developed a phenotype inheritance mathematical formula such that if offspring phenotype is inherited from parent with probability u (e.g. if parent's phenotype was E then an offspring will also inherit the E phenotype with a probability *u*). In case the offspring does not inherit the parental phenotype then it acquires one of the remaining phenotypes with a remaining probability *1-u* that is based upon (i) the number of transitions required to move from the parental phenotype to the given offspring phenotype and (ii) each transition is equally probable with any other transition of equal number of steps. The sum of all possible transitions should add to 1. For example a transition from Prolific to non-Prolific requires one step. A transition from Early to Late requires two steps, Early to Mid followed by Mid to Late. A transition from EP to LnP requires three steps, Prolific to non-Prolific, Early to Mid, and Mid to Late. Further we are not looking at one phenotype but a product of 2. Assume P(E-E)=P(M-M)=P(L-L)=*u_1_* similarly P(P-P)=(nP-nP)=*u_2_* (we have assumed for simplicity that *u_1_* = *u_2_* = *u*). It then follows:

Breeding timing transitions

|  | Offspring | | |
| --- | --- | --- | --- |
| Parent | E | M | L |
| E | u | (1- u)*2/3 | (1 – u)/3 |
| M | (1 – u)/2 | u | (1 – u)/2 |
| L | (1 – u)/3 | (1 – u)*2/3 | u |

Fecundity transitions

|  | Offspring | |
| --- | --- | --- |
| Parent | P | nP |
| P | u | (1 – u) |
| nP | (1 – u) | u |

Combined transitions:

|  | Offspring | | | | | |
| --- | --- | --- | --- | --- | --- | --- |
| Parent | EP | EnP | MP | MnP | LP | LnP |
| EP | u * u | u * (1 – u) | u * (1- u)*2/3 | (1 – u) * (1- u)*2/3 | u * (1 – u)/3 | (1 – u) * (1 – u)/3 |
| EnP | u * (1 – u) | u * u | (1 – u) * (1- u)*2/3 | u * (1- u)*2/3 | (1 – u) * (1 – u)/3 | u * (1 – u)/3 |
| MP | u * (1 – u)/2 | (1 – u) * (1 – u)/2 | u * u | u * (1 – u) | u * (1 – u)/2 | (1 – u) * (1 – u)/2 |
| MnP | (1 – u) * (1 – u)/2 | u * (1 – u)/2 | u * (1 – u) | u * u | (1 – u) * (1 – u)/2 | u * (1 – u)/2 |
| LP | u * (1 – u)/3 | (1 – u) * (1 – u)/3 | u * (1 – u)*2/3 | (1 – u) * (1 – u)*2/3 | u * u | u * ( 1 – u) |
| LnP | (1 – u) * (1 – u)/3 | u * (1 – u)/3 | (1 – u) * (1 – u)*2/3 | u * (1 – u)*2/3 | u * (1 – u) | u * u |

Simplified becomes

|  | Offspring | | | | | |
| --- | --- | --- | --- | --- | --- | --- |
| Parent | EP | EnP | MP | MnP | LP | LnP |
| EP | u^2^ | u * (1 – u) | u * (1- u)*2/3 | (1 – u)^2^ * 2/3 | u * (1 – u)/3 | (1 – u)^2^ /3 |
| EnP | u * (1 – u) | u^2^ | (1 – u)^2^ * 2/3 | u * (1- u) * 2/3 | (1 – u)^2^ /3 | u * (1 – u)/3 |
| MP | u * (1 – u)/2 | (1 – u)^2^ /2 | u^2^ | u * (1 – u) | u * (1 – u)/2 | (1 – u)^2^ /2 |
| MnP | (1 – u)^2^ /2 | u * (1 – u)/2 | u * (1 – u) | u^2^ | (1 – u)^2^ /2 | u * (1 – u)/2 |
| LP | u * (1 – u)/3 | (1 – u)^2^ /3 | u * (1 – u)*2/3 | (1 – u)^2^ * 2/3 | u^2^ | u * ( 1 – u) |
| LnP | (1 – u)^2^ /3 | u * (1 – u)/3 | (1 – u)^2^ * 2/3 | u * (1 – u)*2/3 | u * (1 – u) | u^2^ |

and

|  | Offspring | | | | | |
| --- | --- | --- | --- | --- | --- | --- |
| Parent | EP | EnP | MP | MnP | LP | LnP |
| EP | u^2^ | u – u^2^ | (u – u^2^)* 2/3 | (1 – 2u + u^2^) * 2/3 | (u – u^2^) /3 | (1 – 2u + u^2^) /3 |
| EnP | u – u^2^ | u^2^ | (1 – 2u + u^2^) * 2/3 | (u – u^2^)* 2/3 | (1 – 2u + u^2^) /3 | (u – u^2^) /3 |
| MP | (u – u^2^)/2 | (1 – 2u + u^2^) /2 | u^2^ | u – u^2^ | (u – u^2^)/2 | (1 – 2u + u^2^) /2 |
| MnP | (1 – 2u + u^2^) /2 | (u – u^2^)/2 | u – u^2^ | u^2^ | (1 – 2u + u^2^) /2 | (u – u^2^)/2 |
| LP | (u – u^2^) /3 | (1 – 2u + u^2^) /3 | (u – u^2^)* 2/3 | (1 – 2u + u^2^) * 2/3 | u^2^ | u – u^2^ |
| LnP | (1 – 2u + u^2^) /3 | (u – u^2^) /3 | (1 – 2u + u^2^) * 2/3 | (u – u^2^)* 2/3 | u – u^2^ | u^2^ |

The sum of each row adds to 1 for any u.

For example EP row = u^2^ – u^2^ – 2u^2^/3 + 2u^2^/3-u^2^/3+u^2^/3+u+2u/3-4u/3+u/3-2u/3+2/3+1/3=1

In order to illustrate this example numerically we implemented it in the model using phenotype inheritance data from a published study. A study examining the probability of an offspring inheriting parent's phenotype reports that this probability was ≈ 0.43 or 43% . By replacing u = 0.66 in the last version of the above described calculations we get the phenotype inheritance probabilities used in the model listed in Table 1 below:

**Table 1**. Summary of all phenotype inheritance probabilities used in the model

|  | EP | EnP | MP | MnP | LP | LnP | Total |
| --- | --- | --- | --- | --- | --- | --- | --- |
| EarlyProlific | 43 | 23 | 15 | 7 | 8 | 4 | 100 |
| EarlyNonProlific | 23 | 43 | 7 | 15 | 4 | 8 | 100 |
| MidProlific | 11.5 | 5.5 | 43 | 23 | 11.5 | 5.5 | 100 |
| MidNonProlific | 5.5 | 11.5 | 23 | 43 | 5.5 | 11.5 | 100 |
| LateProlific | 8 | 4 | 15 | 7 | 43 | 23 | 100 |
| LateNonProlific | 4 | 8 | 7 | 15 | 23 | 43 | 100 |

**References**

1. Postma E, Van Noordwijk A (2005) Genetic variation for clutch size in natural populations of birds from a reaction norm perspective. Ecology 86: 2344-2357.
