# Supplementary material for: Integrating Evolution into Ecological Modelling: Accommodating Phenotypic Changes in Agent Based Models
Source: PLoS One. 2013 Aug 5;8(8):e71125. doi: 10.1371/journal.pone.0071125 (PMC3733718; doi:10.1371/journal.pone.0071125)
Supplement: Supplement S3 — Detailed results of linear mixed effects models per phenotype. (DOC) [file pone.0071125.s004.doc]

**Supplement 3**

*Detailed results of linear mixed effects models per phenotype*

Coefficients of linear mixed effects models of individuals per phenotype (dependent variable). The model structure included total prey availability (*mean*), window of prey availability (*var*), as fixed effects, peak week of prey availability during the breeding season (peak2, ..., peak6) as fixed factor, and year as a random effect (see section 'Analysis' for details). A different panel than the one on Figure 3 is listed below: On Figure 3 in the main text model coefficients are plotted for all phenotypes on the same scale while on the one below on an unequal scale so that model coefficients of late phenotypes are more visible.

| **Phenotype: EP** | |  |  |  |  |
| --- | --- | --- | --- | --- | --- |
|  | AIC | BIC | logLik |  |  |
|  | 100520.2 | 100587.1 | -50250.1 |  |  |
|  | Value | Std.Error | DF | t-value | p-value |
| (Intercept) | -615.535 | 63.92389 | 5693 | -10.4656 | <.0001 |
| var | -27.8802 | 2.4263 | 5693 | -11.7176 | <.0001 |
| mean | 637.2696 | 1.91767 | 5693 | 378.6712 | <.0001 |
| peak2 | -341.472 | 47.23957 | 5693 | -8.1857 | <.0001 |
| peak3 | -831.239 | 47.42364 | 5693 | -19.7506 | <.0001 |
| peak4 | -1598.72 | 46.80329 | 5693 | -35.5934 | <.0001 |
| peak5 | -1605.71 | 47.35463 | 5693 | -36.0227 | <.0001 |
| peak6 | -1691.72 | 46.73696 | 5693 | -36.7931 | <.0001 |
| **ANOVA** |  |  |  |  |  |
|  | numDF | denDF | F-value | p-value |  |
| (Intercept) | 1 | 5693 | 1411616 | <.0001 |  |
| var | 1 | 5693 | 163.3 | <.0001 |  |
| mean | 1 | 5693 | 143903.2 | <.0001 |  |
| peak | 5 | 5693 | 508.9 | <.0001 |  |

| **Phenotype EnP** | |  |  |  |  |
| --- | --- | --- | --- | --- | --- |
|  | AIC | BIC | logLik |  |  |
|  | 100353 | 100420 | -50166.5 |  |  |
|  | Value | Std.Error | DF | t-value | p-value |
| (Intercept) | 5.831627 | 63.03965 | 5693 | 0.10221 | 0.9186 |
| var | 21.42279 | 2.39274 | 5693 | 9.4875 | <.0001 |
| mean | 426.7118 | 1.89114 | 5693 | 228.6793 | <.0001 |
| peak2 | 581.4219 | 46.58612 | 5693 | 13.10065 | <.0001 |
| peak3 | -328.274 | 46.76764 | 5693 | -7.32622 | <.0001 |
| peak4 | -1174.04 | 46.15587 | 5693 | -26.2144 | <.0001 |
| peak5 | -1116.95 | 46.69959 | 5693 | -24.9791 | <.0001 |
| peak6 | -1142.85 | 46.09046 | 5693 | -26.9262 | <.0001 |
| **ANOVA** |  |  |  |  |  |
|  | numDF | denDF | F-value | p-value |  |
| (Intercept) | 1 | 5693 | 615390.7 | <.0001 |  |
| var | 1 | 5693 | 69.9 | <.0001 |  |
| mean | 1 | 5693 | 52551.3 | <.0001 |  |
| peak | 5 | 5693 | 557 | <.0001 |  |

| **Phenotype MP** | |  |  |  |  |
| --- | --- | --- | --- | --- | --- |
|  | AIC | BIC | logLik |  |  |
|  | 92451.61 | 92518.61 | -46215.8 |  |  |
|  | Value | Std.Error | DF | t-value | p-value |
| (Intercept) | -386.121 | 32.6327 | 5693 | -10.8797 | <.0001 |
| var | 42.46938 | 1.23861 | 5693 | 32.32291 | <.0001 |
| mean | 112.5662 | 0.97896 | 5693 | 112.6897 | <.0001 |
| peak2 | -138.029 | 24.11547 | 5693 | -5.69276 | <.0001 |
| peak3 | 584.317 | 24.20944 | 5693 | 22.50398 | <.0001 |
| peak4 | 281.1805 | 23.89275 | 5693 | 10.82845 | <.0001 |
| peak5 | -58.7063 | 24.17421 | 5693 | -2.34808 | 0.0189 |
| peak6 | -65.7302 | 23.85889 | 5693 | -2.52187 | 0.0117 |
| **ANOVA** |  |  |  |  |  |
|  | numDF | denDF | F-value | p-value |  |
| (Intercept) | 1 | 5693 | 177248.1 | <.0001 |  |
| var | 1 | 5693 | 1022.74 | <.0001 |  |
| mean | 1 | 5693 | 12658.9 | <.0001 |  |
| peak | 5 | 5693 | 232.98 | <.0001 |  |

| **Phenotype MnP** | |  |  |  |  |
| --- | --- | --- | --- | --- | --- |
|  | AIC | BIC | logLik |  |  |
|  | 91280.67 | 91347.66 | -45630.3 |  |  |
|  | Value | Std.Error | DF | t-value | p-value |
| (Intercept) | -498.656 | 29.59886 | 5693 | -15.5554 | <.0001 |
| var | 46.51874 | 1.123456 | 5693 | 38.6224 | <.0001 |
| mean | 92.03592 | 0.887943 | 5693 | 94.52192 | <.0001 |
| peak2 | -50.4098 | 21.87347 | 5693 | -2.23859 | 0.0252 |
| peak3 | 69.72619 | 21.9587 | 5693 | 3.09443 | 0.002 |
| peak4 | 720.1649 | 21.67146 | 5693 | 30.30863 | <.0001 |
| peak5 | 402.4707 | 21.92675 | 5693 | 17.89388 | <.0001 |
| peak6 | 160.8651 | 21.64075 | 5693 | 6.96156 | <.0001 |
| **ANOVA** |  |  |  |  |  |
|  | numDF | denDF | F-value | p-value |  |
| (Intercept) | 1 | 5693 | 134140.3 | <.0001 |  |
| var | 1 | 5693 | 1517.98 | <.0001 |  |
| mean | 1 | 5693 | 8907.16 | <.0001 |  |
| peak | 5 | 5693 | 314 | <.0001 |  |

| **Phenotype LP** | |  |  |  |  |
| --- | --- | --- | --- | --- | --- |
|  | AIC | BIC | logLik |  |  |
|  | 78384.25 | 78451.24 | -39182.1 |  |  |
|  | Value | Std.Error | DF | t-value | p-value |
| (Intercept) | -129.272 | 10.10504 | 5693 | -12.2827 | <.0001 |
| var | 6.916209 | 0.383547 | 5693 | 17.53051 | <.0001 |
| mean | 16.14677 | 0.303143 | 5693 | 47.99769 | <.0001 |
| peak2 | -1.62547 | 7.467595 | 5693 | -0.20259 | 0.8395 |
| peak3 | -24.1753 | 7.496692 | 5693 | -2.8717 | 0.0041 |
| peak4 | 39.39697 | 7.398628 | 5693 | 5.1402 | <.0001 |
| peak5 | 116.2956 | 7.485784 | 5693 | 14.24935 | <.0001 |
| peak6 | 68.91842 | 7.388142 | 5693 | 9.12568 | <.0001 |
| **ANOVA** |  |  |  |  |  |
|  | numDF | denDF | F-value | p-value |  |
| (Intercept) | 1 | 5693 | 25478.66 | <.0001 |  |
| var | 1 | 5693 | 325.146 | <.0001 |  |
| mean | 1 | 5693 | 2289.868 | <.0001 |  |
| peak | 5 | 5693 | 85.075 | <.0001 |  |

| **Phenotype LnP** | |  |  |  |  |
| --- | --- | --- | --- | --- | --- |
|  | AIC | BIC | logLik |  |  |
|  | 76719.79 | 76786.78 | -38349.9 |  |  |
|  | Value | Std.Error | DF | t-value | p-value |
| (Intercept) | -106.365 | 8.796282 | 5693 | -10.9986 | <.0001 |
| var | 5.804108 | 0.333872 | 5693 | 15.63988 | <.0001 |
| mean | 13.8773 | 0.263882 | 5693 | 46.7098 | <.0001 |
| peak2 | 0.429149 | 6.500427 | 5693 | 0.06029 | 0.9519 |
| peak3 | -19.8415 | 6.525756 | 5693 | -2.74089 | 0.0061 |
| peak4 | -29.3037 | 6.440392 | 5693 | -4.15364 | <.0001 |
| peak5 | -2.24414 | 6.51626 | 5693 | -0.30597 | 0.7596 |
| peak6 | 93.31547 | 6.431264 | 5693 | 14.04769 | <.0001 |
| **ANOVA** |  |  |  |  |  |
|  | numDF | denDF | F-value | p-value |  |
| (Intercept) | 1 | 5693 | 21579.76 | <.0001 |  |
| var | 1 | 5693 | 258.951 | <.0001 |  |
| mean | 1 | 5693 | 2172.767 | <.0001 |  |
| peak | 5 | 5693 | 87.486 | <.0001 |  |
